# Tickborne Relapsing Fever in Southern Iran, 2011–2013

**DOI:** 10.3201/eid2106.141715

**Published:** 2015-06

**Authors:** Saied Reza Naddaf, Behnaz Ghazinezhad, Mohammad Mehdi Sedaghat, Hossein Masoumi Asl, Sally Jane Cutler

**Affiliations:** Pasteur Institute of Iran, Tehran, Iran (S.R. Naddaf, B. Ghazinezhad);; Tehran University of Sciences, Tehran (M.M. Sedaghat);; Ministry of Health and Medical Education, Tehran (H.M. Asl);; University of East London, London, United Kingdom (S.J. Cutler)

**Keywords:** Relapsing fever Borrelia, Borrelia duttonii, Borrelia recurrentis, Borrelia persica, Borrelia microti, tickborne relapsing fever, spirochete, bacteria, Iran, vector-borne infections, ticks

**To the Editor:** Tickborne relapsing fever (TBRF) is endemic in Iran; >1,400 cases were confirmed in 19 provinces during 1997–2006 (*1*). In the western, northwestern, and foothill regions of the Alborz Mountains, the Argasid soft tick *Ornithodoros tholozani* is commonplace and accounts for ≈60% of TBRF cases attributed to *Borrelia persica*. However, in central and western Iran, *O. tholozani* and *B. microti*–infected *O. erraticus* ticks coexist (*1**,**2*). Two other *Borrelia* species, *B. latyschewii* and *B. baltazardi,* have also been described in northeastern and northwestern Iran (*3**,**4*), but no recent human infections with these species have been documented. Cases of TBRF occurring in southern Iran have presumably been caused by *B. microti* because its tick vector, *O. erraticus,* predominates in this region.

Relapsing fever infections in Hormozgan Province in southern Iran are commonly identified during routine checks for malaria. During 2011–2013, blood samples were obtained from 14 febrile patients referred to medical centers in Jask and Rodan in Hormozgan Province (Technical Appendix Figure). Informed verbal consent was obtained from all participants, and the ethical committee of Pasteur Institute of Iran approved the project. Patients seeking care had fever and >1 sign or symptom, such as headache, chills, sweating, or fatigue. Six patients reported recurrent fever and generalized muscle and joint pain. Each patient lived in a local tent, called a kapar, or in a brick or concrete-block house.

Thick and thin blood smears were prepared from blood samples, stained with Giemsa, and examined. None showed malaria parasites; however, spirochetes were observed in thick or thin smears from 3 patients (Technical Appendix Table). Patients whose samples tested positive by microscopy were treated with 500 mg tetracycline every 6 hours for 10 days and became afebrile.

DNA was extracted from patients’ serum samples by using the Miniprep DNA kit (QIAGEN, Hilden, Germany) and screened for borrelia DNA by using real-time PCR; negative and positive control DNA from *B. microti* or *B. persica* was also screened. *Borrelia* spp. DNA was detected in 5 (36%) of 14 serum samples (Technical Appendix Table). Of these 5 samples, 2 were also positive by nested PCR that targeted the intergenic spacer (IGS) region (*5*). The 2 IGS regions were sequenced (ABI-3130XL sequencer; Applied Biosystems, Foster City, CA, USA) in both directions at the Pasteur Institute of Iran. The resulting 539- and 527-bp IGS sequences (GenBank accession nos. KM271987 and KM271988, respectively) were 97% homologous with *B. recurrentis* and *B. duttonii* from Africa (GenBank accession nos. CP000993 and DQ000280, respectively); 96% homologous with *B. microti* from Iran (GenBank accession no. JQ436580); and 92% homologous with *B. crocidurae* from Africa (GenBank accession no. GU350723). A neighbor-joining phylogenetic tree was constructed by using MEGA6 (http://www.megasoftware.net); the 2 IGS sequences clustered into a distinct group separate from *B. microti*, *B. duttonii*, and *B. recurrentis* genotypes (Figure).

**Figure:**
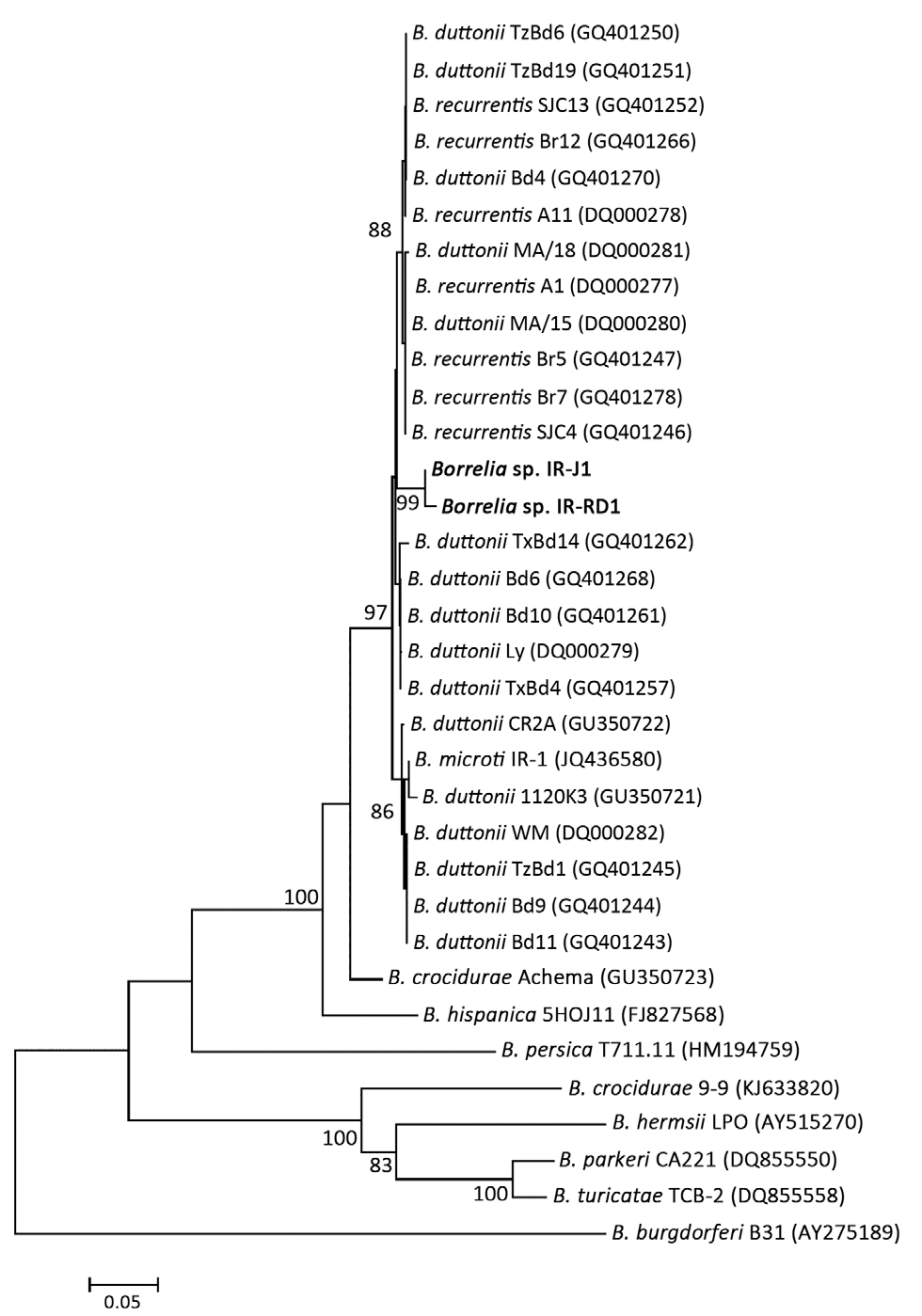
Phylogenetic tree of *Borrelia* spp. strains isolated in Iran, 2014. Constructed on the basis of intergenic spacer sequences, the tree is drawn to scale using evolutionary distance computed using the Jukes-Cantor method in which the units reflect substitutions per site. The final dataset used 587 bp. Numbers at nodes show the level of robustness in a bootstrap test performed with 2,000 replicates; numbers <85 were removed. Scale bar indicates nucleotide substitutions over length analyzed. GenBank accession nos. for nucleotide sequences of IGS from 2 patients (in bold) are KM271987 and KM271988.

*B. microti* was expected to be found because *O. tholozani* ticks that transmit *B. persica* are not seen in southern Iran, but *B. microti*–infected *O. erraticus* ticks have been frequently recovered from rodents’ burrows in the region (*6*). Current molecular data from TBRF borreliae from Iran are limited to 2 isolates of *B. persica* and *B. microti* from *O. tholozani* and *O. erraticus* ticks, respectively (*5**,**7*,*8*). In situ IGS analysis revealed that spirochetes in our analysis had highest homology (97%) with relapsing fever agents from eastern Africa, *B. duttonii* and *B. recurrentis*, followed by *B. microti* (96%) from Iran (*8*). *B. microti* clustered with 1 strain (*B. duttonii*; GenBank accession no. GU350721) and apart from other *B. duttonii* IGS strains, suggesting that this strain may not be *B. duttonii*. The phylogenetic tree separated *B. duttonii* into 4 clades, 2 of which also contained *B. recurrentis*, confirming previous observations (*9*) and providing further support that *B. recurrentis* represents an ecotype of *B. duttonii* rather than a species (*10*). Furthermore, the high level of phylogenetic similarity among borreliae from eastern Africa and Iran indicates that the borreliae in our study might represent ecotype-adapted strains. More sequencing of different genomic markers is required to substantiate or refute this possibility. Lack of GenBank data for the remaining borreliae from Iran, *B. latyschewii* and *B. baltazardi,* prevent exclusion of these species.

Although relapsing fever spirochetes from southern Iran and those from borreliae in Africa have a close phylogenetic similarity, they have different virulence levels and abilities to infect vector and host species. Consequently, deciphering the evolutionary links for these *Borrelia* spp. is of paramount importance and might provide valued insights into host–microbe interactions.

Our report confirms a novel *Borrelia* IGS sequence type detected in situ from 2 relapsing fever patients. This species showed greatest homology with the relapsing fever borreliae from Africa, *B. recurrentis* and *B. duttonii*, but not with *B. microti*, which is transmitted by *O. erraticus* ticks, previously believed to be the only soft tick species in this region. These findings challenge the assumption that TBRF in Iran is attributed to only *B. persica* or *B. microti.*

**Technical Appendix.** Study site, characteristics, and clinical and laboratory findings for patients having positive results for tickborne relapsing fever, Jask and Rodan Counties, Iran, 2011–2013
