# Supplementary material for: Tickborne Relapsing Fever in Southern Iran, 2011–2013
Source: Emerg Infect Dis. 2015 Jun;21(6):1078–80. doi: 10.3201/eid2106.141715 (PMC4451892; doi:10.3201/eid2106.141715)
Supplement: Supplementary file 1 — Technical Appendix. Study site, characteristics, and clinical and laboratory findings for patients having positive results for tickborne relapsing fever, Jask and Rodan Counties, Iran, 2011–2013 [file 14-1715-Techapp-s1.pdf]

Article DOI: http://dx.doi.org/10.3201/eid2106141715

Tickborne Relapsing Fever in Southern Iran, 2011–2013

Technical Appendix

Technical Appendix Table. Characteristics and clinical and laboratory findings for patients having positive results for TBRF by rt-PCR, Jask and Rodan Counties, Iran, 2011–2013*

Patient no.	Sex	Age, y	Occupation	Area, Province	Relapses	Microscopy (count/10 high power fields)	rt-PCR	IGS nested PCR
1	M	22	Unknown	Jask, Hormozgan	Yes	Positive† (2.3)	Positive	Positive
2	M	59	Farmer	Jask, Hormozgan	Yes	Positive† (1)	Positive	Negative
3	M	28	Farmer	Rodan, Hormozgan	Not reported	Positive† (5.2)	Positive	Positive
4	M	18	Unknown	Jask, Hormozgan	Not reported	Negative	Positive	Negative
5	F	25	Housewife	Rodan, Hormozgan	Yes	Negative	Positive	Negative

*TBRF, tickborne relapsing fever, rt-PCR, real-time PCR, IGS, intergenic space;
†Blood from all family members of spirochetemic patients were examined for spirochetes; all had negative results.

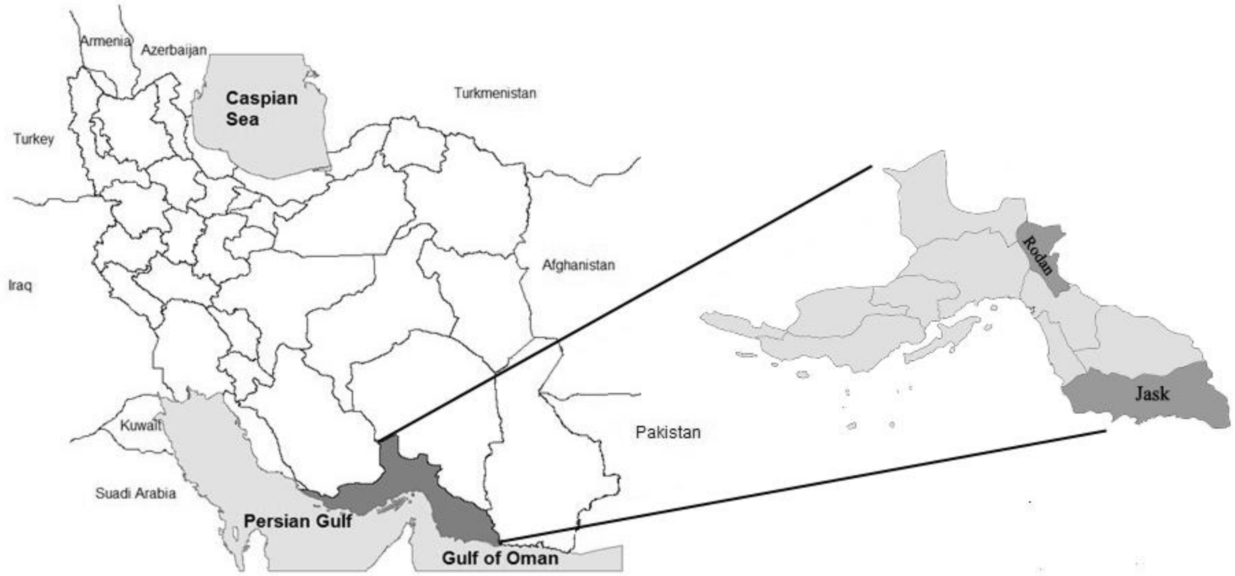

Technical Appendix Figure. Study site (Jask and Rodan Counties) in Hormozgan Province, south Iran, from which patients were recruited.
